# Incidence and Outcome of Invasive Fungal Diseases in Children With Hematological Malignancies and/or Allogeneic Hematopoietic Stem Cell Transplantation: Results of a Prospective Multicenter Study

**DOI:** 10.3389/fmicb.2019.00681

**Published:** 2019-04-16

**Authors:** Thomas Lehrnbecher, Stefan Schöning, Fiona Poyer, Jamina Georg, Andreas Becker, Kathrin Gordon, Andishe Attarbaschi, Andreas H. Groll

**Affiliations:** ^1^Pediatric Hematology and Oncology, Hospital for Children and Adolescents, Johann Wolfgang Goethe-University, Frankfurt, Germany; ^2^Pediatric Hematology and Oncology, St. Anna Children’s Hospital, Medical University of Vienna, Vienna, Austria; ^3^Department of Pediatric Hematology and Oncology, Infectious Disease Research Program, Center for Bone Marrow Transplantation, University Children’s Hospital Münster, Münster, Germany

**Keywords:** child, leukemia, hematopoietic stem cell transplantation, invasive fungal disease, antifungal therapy

## Abstract

**Background:**

Available data on the incidence and outcome of invasive fungal diseases (IFD) in children with hematological malignancies or after allogeneic hematopoietic stem cell transplantation (HSCT) are mostly based on monocenter, retrospective studies or on studies performed prior to the availability of newer triazoles or echinocandins.

**Procedure:**

We prospectively collected clinical data on incidence, diagnostic procedures, management and outcome of IFD in children treated for hematological malignancies or undergoing HSCT in three major European pediatric cancer centers.

**Results:**

A total of 304 children (median age 6.0 years) who underwent 360 therapies (211 chemotherapy treatments, 138 allogeneic HSCTs and/or 11 investigational chemotherapeutic treatments) were included in the analysis. Nineteen children developed proven/probable IFD, mostly due to *Aspergillus* (*n* = 10) and *Candida* spp. (*n* = 5), respectively. In patients receiving chemotherapy, 11 IFDs occurred, all during induction or re-induction therapy. None of these patients died due to IFD, whereas IFD was lethal in 3 of the 8 HSCT recipients with IFD. Significant differences among centers were observed with regard to the use of imaging diagnostics and the choice, initiation and duration of antifungal prophylaxis.

**Conclusion:**

This prospective multicenter study provides information on the current incidence and outcome of IFD in the real life setting. Practice variation between the centers may help to ultimately improve antifungal management in children at highest risk for IFDs.

## Introduction

Available data on the incidence and outcome of invasive fungal diseases (IFD) in children treated for a hematological malignancy or undergoing allogeneic hematopoietic stem cell transplantation (HSCT) are mostly based on single site, retrospective studies or on studies performed prior to the availability of newer compounds such as broad-spectrum triazoles or echinocandins. Single site studies have a high likelihood to be biased by factors such as local epidemiology and local strategies to use available diagnostic tools and to initiate antifungal agents, and therefore, data and conclusions may not be inferred and applied to other centers. In addition, the introduction of newer diagnostic tests and new antifungal agents, as well as the refinement of algorithms in the supportive care in the pediatric setting may have changed the epidemiology and outcome of IFDs over the last decade (Groll et al., 2014; Lehrnbecher et al., 2017a,b). Unfortunately, data from adult studies cannot be transferred to the pediatric population, as children have different underlying malignancies with unique biology, treatments and outcomes, and lack most of the age-dependent comorbidities observed in adults (Sung et al., 2011).

For these reasons, we initiated a prospective study in three major pediatric cancer centers to obtain contemporary data of incidence and outcome of IFDs in children treated for a hematological malignancy or undergoing allogeneic HSCT. We also included specific data on diagnostics and the use of antifungal agents, as they have major impact on the study endpoints.

## Patients and Methods

All children and adolescents less than 18 years of age who were diagnosed between April 1, 2014 and March 31, 2016 with *de novo* acute lymphoblastic leukemia (ALL), acute myeloid leukemia (AML), relapse of acute leukemia, non-Hodgkin lymphoma (NHL), or undergoing allogeneic HSCT were included in this prospective observational study (DRKS00006341). Patients were treated according to Berlin-Frankfurt-Münster (BFM)-based protocols (e.g., AIEOP-BFM ALL 2009, AML-BFM 2012 registry, ALL-REZ BFM registry, or B-NHL 2013) at the University Children’s Hospitals of Frankfurt and Münster, Germany, or at the St. Anna Children’s Hospital, Vienna, Austria. Patients could be enrolled several times in the study if they received different treatments within the study period [e.g., a patient who received chemotherapeutic treatment (usually consisting of several chemotherapy cycles) for *de novo* ALL, followed by relapse chemotherapy and HSCT could potentially be included three times]. The study was reviewed and approved by the local Ethical committees of all three participating centers (Frankfurt: 348/13; Münster: 2014-048-b-S; Vienna: 17-6-2014).

According to current pediatric guidelines, all centers routinely performed diagnostics such as imaging studies in children at highest risk for IFD, e.g., persistently febrile neutropenic patients not responding to broad-spectrum antibiotics after 96 h or patients with any clinical sign or symptom consistent with IFD (Groll et al., 2014; Lehrnbecher et al., 2017b). These patients also received empirical or pre-emptive mold-active antifungal therapy, respectively. Regarding antifungal prophylaxis, no common policy was adopted by the participating centers, and prophylaxis was instituted according to local standard operating procedures.

Data collection was performed using an electronic database (secuTrial^®^), and included demographic data, disease characteristics, data on laboratory diagnostic studies and imaging as well as information on antifungal drug use. Data were collected for each cycle of intensive chemotherapy, defined by the first day of chemotherapy until the start of the next cycle of chemotherapy, and for patients undergoing HSCT from the start of the conditioning regimen until at least day +100. Last follow-up was after 12 months after the end of intensive chemotherapy or the date of allogeneic HSCT and included the current status on relapse/disease-free survival, death (including cause of death) and, in case of the occurrence of an IFD, the outcome of this infection.

### Definitions

Fever was defined as temperature higher than 38.5°C once or between 38 and 38.5°C twice within a 4-h interval, and neutropenia as an absolute neutrophil count ≤500/mm (Lehrnbecher et al., 2017b). Acute and chronic graft-vs.-host disease (GVHD) was defined according to NIH criteria (Filipovich et al., 2005). Invasive fungal disease was defined as proven, probable, and possible infection according to the revised definitions by the EORTC/MSG consensus group (De Pauw et al., 2008). In brief, proven IFD required detection of a fungus by culture in blood or an otherwise sterile compartment, or histopathological evidence of fungal elements in affected tissue. Probable IFD was defined by the presence of host factors (e.g., severe and prolonged neutropenia, allogeneic HSCT), clinical criteria [e.g., lower respiratory tract infection with computerized tomography (CT) imaging demonstrating lesions suggestive of an IFD], and mycological criteria [e.g., culture of a mold in sputum or broncho-alveolar lavage (BAL), detection of galactomannan (GM) in serum (optical density index of >1.0 (one sample) or >0.5 (two samples) or BAL (cut-off 1.0)]. Positive testing by PCR was not included as criterion of probable IFD. Patients with appropriate host factors and with sufficient clinical evidence consistent for IFD, but for whom there was no mycological support, were categorized as patients with possible IFD (De Pauw et al., 2008).

The response to antifungal treatment was defined on the basis of modified criteria provided previously (Denning et al., 2008). The resolution of all clinical signs and symptoms attributable to IFD together with nearly complete radiographic resolution of all lesions, where present, was defined as complete remission; major clinical improvement together with at least 50% improvement of radiological signs, where present, was defined as partial response, whereas less than 50% improvement was defined as stable disease, and progression of IFD and death as failure.

### Statistical Analysis

For the analysis, GraphPad Prism (version 5.04 for Windows; GraphPad Software, San Diego, CA, United States) was used. Chi-square test, Fishert’s exact test, and unpaired *t* test were performed, as applicable. A two-sided *P* ≤ 0.05 was considered to be statistically significant.

## Results

### Patients’ Characteristics

A total of 304 patients were included in the study. Hundred-and-seven of the patients were treated in Frankfurt, 81 in Münster, and 116 patients received chemotherapy and/or were undergoing allogeneic HSCT in Vienna (Table 1). The median age (range) of the 190 boys (62.5%) and 114 girls (37.5%) at diagnosis was 6.0 years (1 month to 17.9 years) without significant differences between the three centers.

**Table 1:** Characteristics of 304 patients receiving 360 treatments [chemotherapeutic treatment, hematopoietic stem cells transplantation (HSCT), or experimental chemotherapeutic treatment] in one of the study centers.

Centers	Frankfurt	Münster	Vienna	Total
Number of patients	107	81	116	304
Male/female	71/36	52/29	67/49	190/114
Age (median, range in years)	5.9 (0.2–17.8)	6.5 (0.1–17.9)	6.5 (0.1–17.9)	6.0 (0.1–17.9)
Total number of treatments	127^∗^	98^∗^	135^∗^	360^∗^
BFM-based chemotherapeutic treatments ^∗∗^	59	59	93	211
Number of chemotherapy cycles (total)	198	192	371	761
Average number (range) of chemotherapy cycles per patient	3.4 (2–8)	3.2 (1–8)	4.0 (2–8)	3.8 (1–8)
Underlying malignancy (patients with BFM-based chemotherapy)				
ALL (total)	40	33	60	133
ALL-SR	16	15	21	52
ALL-MR	17	11	22	50
ALL-HR	7	7	17	31
ALL relapse	3	3	12	18
AML	6	9	8	23
AML relapse	0	3	2	5
NHL	10	11	11	32
Experimental chemotherapeutic treatments^∗∗^	8	2	1	11
Hematopoietic stem cell transplantation	60	37	41	138
Underlying disease				
ALL/ALL relapse	23	14	15	52
AML/AML relapse	10	8	8	26
MDS/AA	8	8	8	24
Lymphoma	4	4	4	12
Immunodeficiency	9	1	4	14
Sickle cell disease	2	0	0	2
Thalassemia	1	0	1	2
Other^∗∗∗^	3	2	1	6

*^∗^ A total of 34, 8, and 2 patients, respectively, received more than one treatment and were included twice, three times or four times in the study. ^∗∗^Chemotherapeutic treatments were divided in treatment regimens based on current Berlin-Frankfurt-Münster (BFM) trials and in treatment regimens with experimental character in the pediatric setting such as blinatumomab or bortezomib (“experimental chemotherapeutic treatment”). Experimental chemotherapeutic treatment was excluded from further analysis. ^∗∗∗^Includes two patients each with rhabdomyosarcoma and hemophagocytic syndrome, and one patient each with Blackfan-Diamond anemia and chronic myeloid lymphoma. ALL, acute lymphoblastic leukemia; ALL-SR, ALL-standard-risk; ALL-MR, ALL-medium-risk; ALL-HR, ALL-high-risk; AML, acute myeloid leukemia; NHL, non-Hodgkin lymphoma; HSCT, hematopoietic stem cell transplantation; MDS, myelodysplastic syndrome; AA, aplastic anemia.*

The 304 patients received a total of 360 treatments which included 211 BFM-based chemotherapy treatments (consisting of several chemotherapy courses in most patients), 11 investigational chemotherapy treatments which were defined as a therapeutic regimen using compounds with limited experience in children such as blinatumomab, inotuzumab, or bortezomib, and 138 allogeneic HSCTs (Table 1). A total of 34 patients were included twice in the study: 24 and 5 patients received chemotherapy or investigational chemotherapy prior to HSCT, respectively, and 5 patients were undergoing HSCT twice. Eight patients were included three times, two patients four times. The 11 investigational chemotherapeutic treatments were excluded from further analysis.

The most common underlying malignancy of patients receiving chemotherapy was a new diagnosis of ALL (*n* = 133), whereas less patients were treated for NHL (*n* = 32), AML (*n* = 23), or relapsed leukemia (*n* = 22). Most allogeneic HSCT recipients were treated for *de novo* or relapsed ALL and AML, respectively (*n* = 52 and *n* = 26) (Table 1). One year after the end of intensive chemotherapy/HSCT, 281 out of the 304 patients were alive (92.4%).

### Proven, Probable, and Possible IFDs

Overall, 10 proven and 9 probable IFDs were diagnosed in 12 boys and 7 girls (overall incidence rate: 6.3%) (Tables 2, 3). *Aspergillus* and *Candida* spp. were seen in 10 and 5 patients, respectively, and one patient each suffered from fusariosis and co-infection with *Rhizopus arrhizus* and *A. fumigatus*, respectively. In 2 patients, invasive mold infection was diagnosed on the basis of histopathological but no further mycological evidence.

**Table 2:** Patients suffering from proven invasive fungal disease.

Center	Frankfurt	Frankfurt	Vienna	Vienna	Vienna	Vienna	Vienna	Vienna	Vienna	Vienna
Sex/age (years)	m/11.3	m/4.7	m/1.8	m/11.5	f/16.5	f/14.9	m/16.3	m/14.6	f/9.5	m/16.0
Malignancy (risk group)	Rhabdo	CGD	ALL (SR)	ALL (SR)	ALL (HR)	AML	AML	ALL (relapse)	AML (relapse)	AML (relapse)
Chemo/ HSCT	HSCT	HSCT	Chemo	Chemo	Chemo	HSCT	HSCT	Chemo	Chemo	Chemo
Time point	Post-engraft	Pre-engraft	Induction	Re-induction	Induction	Pre-engraft	pre-engraft	Induction	Induction	Induction
Risk factors	S	N	N, S	S	N, S	N	N	N	N	N
Pathogen	*C. krusei*	*A. fumigatus*	*C. albicans*	A. *fum./Rhizopus arrhizus*	mold	*Fusarium* spp.	*C. krusei*	mold	*C. albicans*	*C. krusei*
Site	Blood (culture)	Lung, brain biopsy (culture)	Blood (culture)	Sinus/maxilla surgery (culture)	Lung biopsy (histology)	Eye/blood/skin (culture skin and blood)	Blood (culture)	Lung biopsy (histology)	Blood (culture)	Blood (culture)
Prophylaxis	Antimold (LAMB)	Antimold (Vori)	No	No	Antimold (LAMB/Vori^∗^)	Antimold (Posa)	No	Antimold (Vori)	Antimold (Vori)	Antimold (Posa)
Outcome IFD	CR	Death	CR	PR	CR	CR	Death	CR	CR	CR
Outcome	Death unrelated to IFD	Death due to IFD	Alive	Alive	Alive	Alive	Death due to IFD	Death unrelated to IFD	Death unrelated to IFD	Death unrelated to IFD

*F, female; m, male; AML, acute myeloid leukemia; ALL, acute lymphoblastic leukemia; ALL-SR, ALL-standard-risk group; ALL-HR, ALL-high-risk group; rhabdo rhabdomyosarcoma; CGD, chronic granulomatous disease; chemo chemotherapy; HSCT, hematopoietic stem cell transplantation; engraft engraftment; N, prolonged neutropenia (<500 neutrophils/mm^3^ >10 days); S use of corticosteroids at a mean minimum dose of 0.3 mg/kg/day prednisone equivalent for more than 3 weeks; LAMB, liposomal amphotericin B; Vori voriconazole; Posa posaconazole; CR, complete remission; PR, partial remission; IFD, invasive fungal disease.*

**Table 3:** Patients suffering from probable invasive fungal disease.

Center	Frankfurt	Frankfurt	Frankfurt	Münster	Münster	Münster	Münster	Münster	Münster
Sex/age (years)	f/16.9	m/9.5	f/16.8	m/17.4	f/15.3	m/3.9	m/2.3	m/17.1	m/13.3
Malignancy (risk group)	AML	ALL	AML	ALL (HR)	ALL	AML	ALL (MR)	ALL (SR)	AA
Chemo/HSCT	HSCT	HSCT	Chemo	Chemo	HSCT	Chemo	Chemo	Chemo	HSCT
Time point	Post-engraft	Post-engraft	Re-induction	Induction	Pre-engraft	Induction	Induction	Re-induction	Pre-engraft
Risk factors	S, CSA	S	N	N, S	N	N	N, S	S	N
Pathogen	Aspergillus	Aspergillus	Aspergillus	Aspergillus	Aspergillus	Aspergillus	Aspergillus	Aspergillus	Aspergillus
Site	Lung (imaging, serum GM)	Lung (imaging, serum GM)	Lung (imaging, serum GM, and GM in BAL)	Lung/brain (imaging, serum GM, and GM in BAL)	Lung (imaging, BAL culture)	Lung (imaging, serum GM)	Lung (imaging, serum GM)	Lung (imaging, serum GM)	Lung (imaging, serum GM)
Prophylaxis	Antimold (LAMB, Vori^∗^)	Antimold (Vori)	Antimold (Mica, Posa^∗^)	No	Antimold (Vori)	Antimold (LAMB)	Antimold (LAMB)	No	Antimold (Vori)
Outcome IFD	CR	PR	PR	CR	Death	PR	CR	CR	SD
Outcome	Alive	Death unrelated to IFD	Alive	Alive	Death due to IFD	Alive	Alive	Alive	Alive

*f, female; m, male; AML, acute myeloid leukemia; ALL, acute lymphoblastic leukemia; ALL-SR, ALL-standard-risk group; ALL-MR, ALL-medium-risk group; ALL-HR, ALL-high-risk group; AA, aplastic anemia; chemo chemotherapy; HSCT, hematopoietic stem cell transplantation; engraft engraftment; N, prolonged neutropenia (<500 neutrophils/mm^3^ >10 days); S use of corticosteroids at a mean minimum dose of 0.3 mg/kg/day prednisone equivalent for more than 3 weeks; GM, galactomannan; BAL, broncho-alveolar lavage; LAMB, liposomal amphotericin B; Vori voriconazole; Posa posaconazole; Mica micafungin; CR, complete remission; PR, partial remission; SD, stable disease; IFD, invasive fungal disease.*

Eleven IFDs occurred during the 211 chemotherapy treatments [6 IFDs in ALL patients [3 ALL-standard-risk (SR), 1 ALL-medium-risk (MR), 2 ALL-high-risk (HR)], 1 in a patient with ALL relapse, and 2 each in patients with AML and AML relapse]. Incidence rates of IFD were 4.5% for ALL, 8.7% for AML, and 13.6% for leukemia relapse, whereas no IFD occurred in patients with NHL. The median age of the patients developing an IFD during chemotherapeutic treatment was 16.0 years (range, 1.8–17.4), and patients with invasive mold infection (*n* = 8) were significantly older than those without IFD [15.6 (2.3–17.4) vs. 5.3 (0.2–17.9); *P* = 0.002]. All affected patients had at least one risk factor such as prolonged neutropenia (<500 neutrophils/mm^3^ >10 days) or prolonged use of therapeutic doses of corticosteroids. In patients receiving a chemotherapeutic treatment, all IFDs occurred during the phases of induction or re-induction. Seven patients had received mold-active antifungal prophylaxis prior to IFD, whereas 4 patients (3 ALL-SR, 1 ALL-HR) had not received antifungal prophylaxis. In all patients with IFD occurring during chemotherapy, complete or partial remission could be achieved. Three of the patients died due to reasons unrelated to their IFD.

A total of 8 IFDs occurred in HSCT patients (four each during the pre- and post-engraftment period) (incidence 5.8%). The median age (range) of the patients with IFD was 14.1 years (4.7–17.1). Three of the HSCT patients with IFD survived, whereas 3 patients died due to IFD (two patients with invasive aspergillosis, 1 patient with invasive *Candida* infection), and 2 patients died due to unrelated reasons.

Out of the 13 boys and 10 girls with possible IFDs, 12 patients had received chemotherapeutic treatment [median age (range) 8.4 years (2.4–15.9)], and 11 had undergone allogeneic HSCT [median age (range) 9.4 years (0.5–17.8)] (Supplementary Table). All possible IFDs were diagnosed according to host factors and clinical signs and symptoms in the lung, and all possible IFDs in patients receiving chemotherapy occurred during the phase of induction or re-induction (3 patients and 1 patient with ALL and ALL relapse, 4 patients and 2 patients with AML and AML relapse, and 2 patients with NHL, respectively). In patients undergoing HSCT, IFD occurred in 6 patients in the pre-engraftment and in 5 patients in the post-engraftment period, respectively. Notably, 3 of the 5 patients with possible IFD during the post-engraftment period had GVHD grades III or IV. None of the patients with possible IFD died due to the fungal infection, whereas 4 HSCT patients died due to reasons unrelated to IFD. One patient was lost for follow-up.

### Diagnostic Procedures in Patients With Suspected IFD

In addition to routine diagnostics during febrile neutropenia such as blood cultures, each of the three participating centers used diagnostic tools to detect IFD such as serum GM or CT scan of the lung, although a significant variation between the sites was seen (Table 4). All centers performed invasive diagnostics such as BAL or biopsy.

**Table 4:** Number of treatments/number of total treatments (chemotherapy and hematopoietic stem cell transplantation, respectively, percentage) in which a diagnostic test was performed at least once in order to detect invasive fungal infection.

Diagnostic tool	Frankfurt	Münster	Vienna	Total	*P*
Galactomannan					
Chemotherapy	16/59 (27)	16/59 (27)	8/93 (9)	40/211 (19)	0.003
Allogeneic HSCT	60/60 (100)^∗^	31/37 (84)	41/41 (100)	132/138 (96)	0.0002
CT^∗∗^	37/119 (31)	52/96 (54)	10/134 (7)	99/349 (28)	<0.0001
BAL^∗∗^	4/119 (3)	4/96 (4)	1/134 (1)	9/349 (3)	ns
Biopsy^∗∗^	4/119 (3)	2/96 (2)	8/134 (6)	14/349 (4)	ns


*^∗^Used as screening method. ^∗∗^Chemotherapeutic treatments and HSCT combined. HSCT, hematopoietic stem cell transplantation; CT, computerized tomography; BAL, broncho-alveolar lavage.*

### Antifungal Prophylaxis and Empiric Therapy

The use of prophylactic antifungals considerably differed between the specific ALL-risk groups, namely ALL-SR, ALL-MR, and ALL-HR. Whereas all three sites used prophylactic antifungals in only a minority of ALL-SR patients, almost all ALL-HR patients who are treated with a more dose-intensive chemotherapy received antifungal prophylaxis (Table 5). Significant differences between the centers were seen in the initiation of antifungal prophylaxis in the ALL-MR group (*P* = 0.019) (Table 5), with Vienna using antifungal prophylaxis in more patients as compared to Frankfurt (15/22 vs. 4/17; *P* = 0.0095). Similar results were seen when combining ALL-SR and ALL-MR patients, who receive almost identical chemotherapeutic regimens (23/43 vs. 7/33; *P* = 0.005). Antifungal prophylaxis in the ALL-SR and ALL-MR patient was given in most patients only during the induction and re-induction phase, and not during consolidation (protocol M) (data not shown). Significant differences between the centers were also noted for the duration of prophylaxis. As compared to Vienna, the cumulative number of days for antifungal prophylaxis in ALL-MR patients was significantly longer in Frankfurt (median, lower/upper quartile; 131 (120–182) vs. 14.5 (5–16); *P* < 0.0001). In addition to the initiation and the duration of antifungal prophylaxis, the use of specific antifungal compounds considerably differed between the sites. For example, the proportion of fluconazole used for antifungal prophylaxis in ALL patients varied between 4 and 21%, of voriconazole between 0 and 72%, of posaconazole between 4 and 33%, of liposomal amphotericin B between 15 and 73%, and that of micafungin between 0 and 20%, respectively (data not shown). In contrast to patients with ALL, the vast majority of patients with *de novo* AML or with relapsed leukemia (both ALL and AML) received antifungal prophylaxis throughout all chemotherapy cycles in all centers.

**Table 5:** Use of antifungal prophylaxis and of empirical antifungal therapy in children with acute lymphoblastic leukemia (ALL).

	Frankfurt		Münster		Vienna		*P*
Patients	n^∗^	%^∗^	n	%	n	%	
ALL-SR (n,%)	16	100	15	100	21	100	
No antifungals	13	81	8	53	13	62	ns
Antifungal prophylaxis	3	19	4	27	8	38	ns
Number of days on prophylaxis (median, upper, and lower quartile)	14.0 (9.0–29.0)		23 (15.5–29)		8 (4.25–22)		ns^∗∗^
Empiric antifungal therapy	0	0	3	20	2	10	ns
Number of days on empirical therapy (individual pts)			7, 8, 9		9, 12		nd^∗∗∗^
ALL-MR (n, %)	17	100	11	100	22	100	
No antifungals	12	71	5	45	7	32	ns
Antifungal prophylaxis	4	24	5	45	15	68	0.019
Number of days on prophylaxis (median, upper, and lower quartile)	131.0 (120.0–182.0)		18.0 (14.5–50)		14.5 (5–16)		<0.0001 F vs. V, ns F vs. M, M vs. V
Empiric antifungal therapy	3	18	2	18	1	5	ns
Number of days on empirical therapy (individual pts)	10, 10, 26		4, 11		5		nd^∗∗∗^
ALL-HR (n, %)	7	100	7	100	17	100	
No antifungals	0	0	1	14	0	0	ns
Antifungal prophylaxis	7	100	6	86	17	100	ns
Number of days on prophylaxis (median, upper, and lower quartile)	148 (43–179)		34.5 (20.75–68.5)		56 (24–63)		0.0038 F vs. V, ns F vs. M, M vs. V
Empiric antifungal therapy	1	14	3	43	4	24	ns
Number of days on empirical therapy (individual pts)	10		3, 10, 40		2, 11, 16, 19		nd^∗∗∗^

*^∗^Due to the fact that patients may receive both antifungal prophylaxis and empiric therapy, the percentage of patients without and with antifungals may exceed 100%. ^∗∗^ ns for any comparison (Frankfurt vs. Münster, Frankfurt vs. Vienna, and Münster vs. Vienna). ^∗∗∗^ Not done due to the small number of patients receiving empirical antifungal treatment. When combining ALL-SR and ALL-MR patients, who receive the almost identical chemotherapeutic regimens, all comparisons were not significant. ALL-SR, ALL-standard-risk group; ALL-MR, ALL-medium-risk group; ALL-HR, ALL-high-risk group; ns, not significant; nd, not done; F, University Hospital Frankfurt; M, University Hospital Münster; V, St. Anna Childrent’s Hospital Vienna; vs, versus.*

No significant differences between the centers were seen in the use of antifungal prophylaxis for patients undergoing allogeneic HSCT. All patients received antifungal prophylaxis, starting at the time of or briefly after the conditioning regimen, and lasting beyond engraftment until cessation of immunosuppression. However, as noted for patients with ALL, the preferred choice of antifungal compounds differed considerably between the centers (data not shown).

Despite significant differences in the initiation, duration and choice of antifungal prophylaxis, there were no major differences between the centers regarding initiation and duration of empirical antifungal therapy. Importantly, the risk of breakthrough infections was not associated with a specific antifungal compound (Tables 2, 3).

## Discussion

Contemporary data on epidemiology, treatment and outcome of IFD in children with hematological malignancies or undergoing allogeneic HSCT are important as they may help to improve antifungal management (Pana et al., 2017). Whereas our data on the incidence of IFDs in the different subgroups corroborate the results of a recent multicenter analysis (Cesaro et al., 2017), several other studies reported on higher incidences (Hovi et al., 2000; Castagnola et al., 2006; Sung et al., 2007; Hale et al., 2010). These differences may be explained not only by local epidemiology, but also by the fact that studies often included “suspected” fungal infections in their analysis. We considered only the 19 patients with proven and probable IFDs for the calculation of incidence rates, but excluded the 23 additional patients with possible IFDs. The relatively high percentage of possible IFD in our analysis as compared to previous data may be due to the fact that rigorous imaging studies were performed if signs or symptoms were indicating the potential presence of an IFD, although microbiological tests remained negative. The information on the use of diagnostic tools for the analysis of the epidemiology of IFD is important, which is supported by the observation that the number of CT scans performed in each center positively correlated with the rate of possible IFD. Similarly, the information whether patients received antifungal prophylaxis or not is essential for the interpretation of reported incidence rates of IFDs, but is unfortunately not reported by all studies.

Although patients with ALL are at a lower risk for IFDs compared to children with AML or leukemia relapse, it is important to analyze this patient population in more detail for several reasons. First, in pediatric cancer patients, this entity comprises the largest group of patients at risk for IFDs, and second, the largest absolute number of IFDs is diagnosed in pediatric ALL patients (Cesaro et al., 2017). Whereas most studies do not discriminate between the different risk groups of children with ALL who receive different treatment intensities which influence the risk for IFDs, Hale et al. (2010) reported on a considerably lower incidence of IFDs in “low-risk” ALL, and no invasive mold infection occurred in these “low-risk” ALL patients. In contrast, we found 3 out of 52 “low-risk” ALL patients with invasive mold infection, who also comprised half of the 6 ALL-patients in whom this infection was diagnosed. Notably, significant differences were observed in ALL-SR and ALL-MR patients in the initiation and duration of antifungal prophylaxis and in the choice of the antifungal compound. For example, as compared to Vienna, antifungal prophylaxis was initiated in significantly less ALL-SR and ALL-MR patients treated in Frankfurt, but, on the other hand, the duration of antifungal prophylaxis was significantly longer in Frankfurt as compared to Vienna. Therefore, one is prompted to speculate that both indication and duration of antifungal prophylaxis could be optimized. Unfortunately, it has not fully been clarified how to predict the individual risk for IFDs in ALL-SR and ALL-MR patients. However, corroborating the findings of a recent meta-analysis (Fisher et al., 2017), our study demonstrates that patients with invasive mold infection were significant older than patients without IFDs, and that all IFDs occurred during phases of chemotherapy phases where patients had profound and prolonged neutropenia or received prolonged high-dose corticosteroid therapy. Based on these findings, the site in Frankfurt has recently modified its antifungal strategy and administers antifungal prophylaxis during the phases of induction and re-induction to all ALL-SR and ALL-MR patients that are older than 10 years. Whether this will decrease the rates of IFDs and will be cost effective is the focus of a planned audit. The wide variety between the centers in the choice of the antifungal compounds reflects the uncertainties in the pediatric setting, in particular in children with ALL. In these patients, the use of broad-spectrum triazoles such as voriconazole (approved for children older than 2 years) or posaconazole (no pediatric label within the European Union) is limited due to the interaction with vinca-alkaloids, which are a cornerstone in the treatment of ALL. On the other hand, amphotericin B formulations and the new class of echinocandins, which are not approved for anti-mold prophylaxis, have to be administered intravenously on a daily basis. Whether intermittent dosing of these compounds may become a suitable option needs to be explored in future studies (Bochennek et al., 2011, 2015).

Our analysis demonstrated a favorable response (CR or PR) in 15 patients (80%), whereas 3 deaths were caused by IFD (15.8%), all of them occurring in the pre-engraftment period of patients undergoing HSCT. The substantially lower mortality relative to the reports of previous, older studies is most likely due to the improvement of supportive care, including newer diagnostic tools and the availability of new, versatile and potent antifungal compounds (Hovi et al., 2000; Castagnola et al., 2006).

We recognize that our study has several limitations, including the lack of uniform operating procedures for diagnostics and management which, however, is inherent to any epidemiological study. Due to the relatively small number of patients with proven and probable IFD we did not perform a multivariate analysis of risk factors. On the other hand, our analysis has unique and important strengths. For example, the prospectively collected data of three major pediatric cancer centers provide information on the current incidence and outcome of IFD in2 the real-life setting outside prospective randomized trials on antifungal management. In addition, different to previous studies, our analysis includes detailed data on the use of diagnostic tests and the use of prophylactic antifungal agents, which is a prerequisite for interpreting the risk of IFDs. Finally, differences between the centers in both the initiation and cessation of antifungal prophylaxis identify an important research gap, and allow a preliminary comparative analysis, although the conclusions might be limited by not pre-defined strategies.

## Conclusion

In conclusion, this prospective multicenter study confirms that children treated for ALL, AML or undergoing HSCT have a significant risk for IFDs, and the risk is individually determined by factors such as age or treatment course. IFD-related mortality was low, in particular in patients receiving chemotherapy. Differences between the sites in the use of imaging diagnostics and antifungal prophylaxis may help to identify approaches to improve the antifungal management in particular of children with ALL, the largest at risk population in pediatric cancer medicine.

## Ethics Statement

The Ethical committees of each participating center (Frankfurt, Münster, Vienna) have reviewed and approved the study (approval numbers 348/13 for Frankfurt, 2014-048-b-S for Münster, and 17-6-201 for Vienna). Patients were included in the analysis if they and/or their caregivers had given written informed consent.

## Author Contributions

All authors have significantly contributed to the manuscript by designing the study (TL, SS, AA, and AG), collecting the data (FP, JG, AB, and KG), and analyzing the data (all authors). All authors have reviewed and agreed upon the manuscript content.

## Conflict of Interest Statement

TL has received research grants from Gilead Sciences, is a consultant to Astellas, Basilea, Gilead Sciences, and Merck/MSD, and served at the speaker’s bureau of Astellas, Gilead Sciences, Merck/MSD, and Pfizer. AA has a consulting and advisory role for Jazz Pharmaceuticals and received travel and accommodations expenses from Jazz Pharmaceuticals, Amgen, and Pfizer. AG has received grants from Gilead, Merck, Sharp and Dohme, Pfizer, and Schering-Plough; is a consultant to Amplyx, Astellas, Basilea, Gilead, Merck, Sharp and Dohme, and Schering-Plough, and served at the speakers’ bureau of Astellas, Basilea, Gilead, Merck, Sharp and Dohme, Pfizer, Schering-Plough, and Zeneus/Cephalon. The remaining authors declare that the research was conducted in the absence of any commercial or financial relationships that could be construed as a potential conflict of interest.
