# Supplementary material for: Incidence and Outcome of Invasive Fungal Diseases in Children With Hematological Malignancies and/or Allogeneic Hematopoietic Stem Cell Transplantation: Results of a Prospective Multicenter Study
Source: Front Microbiol. 2019 Apr 16;10:681. doi: 10.3389/fmicb.2019.00681 (PMC6476895; doi:10.3389/fmicb.2019.00681)
Supplement: Supplementary file 1 [file Table_1.DOCX]

**Supplementary Table:** Characteristics of patients diagnosed with possible invasive fungal disease

| Center | Frankfurt | Frankfurt | Frankfurt | Frankfurt | Münster | Münster | Münster | Münster |
| --- | --- | --- | --- | --- | --- | --- | --- | --- |
| Sex/age (years) | f/0.5 | m/6.4 | m/6.5 | m/15.3 | f/5.2 | m/12.3 | m/17.8 | m/17.2 |
| Malignancy (risk group) | BDA | AML | AML | ALL | ALL (SR) | ALL (HR) | AA | ALL |
| Chemo/HSCT | HSCT | chemo | HSCT | HSCT | chemo | chemo | HSCT | HSCT |
| Time point | pre-engraft | induction | pre-engraft | post-engraft | re-induction | Prot III | pre-engraft | post-engraft |
| Prophylaxis | yes | yes | yes | yes | no | yes | Yes | yes |
| Outcome IFD | CR | CR | CR | CR | PR | CR | CR | CR |
| Outcome | alive | alive | alive | alive | alive | alive | Alive | death unrelated to IFD |

| Center | Münster | Münster | Münster | Münster | Münster | Münster | Münster | Münster |
| --- | --- | --- | --- | --- | --- | --- | --- | --- |
| Sex/age (years) | f/13.0 | m/4.9 | f/2.4 | f/2.7 | m/4.4 | m/15.9 | m/13.1 | m/5.6 |
| Malignancy (risk group) | AML | Immunode-ficiency | AML (relapse) | AML | AML | ALL (relapse) | NHL | NHL |
| Chemo/HSCT | chemo | HSCT | chemo | HSCT | HSCT | chemo | chemo | chemo |
| Time point | re-induction | post-engraft | induction | pre-engraft | post-engraft | induction | re-induction | re-induction |
| Prophylaxis | yes | yes | yes | yes | yes | no | No | no |
| Outcome IFD | CR | PR | PR | PR | PR | PR | CR | CR |
| Outcome | alive | death unrelated to IFD | alive | death unrelated to IFD | lost for follow up | alive | Alive | alive |

| Center | Münster | Münster | Münster | Münster | Münster | Münster | Münster |
| --- | --- | --- | --- | --- | --- | --- | --- |
| Sex/age (years) | f/10.3 | m/5.2 | m/10.9 | f/16.7 | f/17.4 | f/4.8 | f/15.3 |
| Malignancy (risk group) | AML (relapse) | AML | ALL (HR) | ALL | Immunode-ficiency | AML | AML |
| Chemo/HSCT | chemo | chemo | chemo | HSCT | HSCT | chemo | HSCT |
| Time point | induction | induction | Prot III | post-engraft | post-engraft | induction | pre-engraft |
| Prophylaxis | yes | yes | yes | yes | yes | yes | Yes |
| Outcome IFD | CR | CR | CR | CR | PR | CR | CR |
| Outcome | alive | alive | alive | alive | death unrelated to IFD | alive | Alive |

f female

m male

BDA Diamond-Blackfan anemia

AML acute myeloid leukemia

ALL acute lymphoblastic leukemia

ALL-SR ALL-standard-risk group

ALL-HR ALL-high-risk group

AA aplastic anemia

NHL non-Hodgkin lymphoma

chemo chemotherapy

HSCT hematopoietic stem cell transplantation

engraft engraftment

CR complete remission

PR partial remission

IFD invasive fungal disease
